# Left bundle cardiac resynchronization therapy versus left bundle optimized cardiac resynchronization therapy

**DOI:** 10.1016/j.ipej.2026.02.009

**Published:** 2026-02-14

**Authors:** Chinmay Parale, Suresh Kumar Sukumaran, Dinakar Bootla, K.E. Sivavignesh, Ashish Jain, Sridhar Balaguru, Santhosh Satheesh, Avinash Anantharaj, A Shaheer Ahmed, Raja Selvaraj

**Affiliations:** Department of Cardiology, Jawaharlal Institute of Postgraduate Medical Education and Research, Puducherry, India

**Keywords:** Conduction system pacing, Left bundle branch area pacing, Cardiac resynchronization therapy, Echocardiography, Non-ischemic cardiomyopathy

## Abstract

**Background:**

In patients undergoing left bundle branch area pacing for cardiac resynchronization therapy (LB-CRT), addition of a coronary sinus lead i.e. Left bundle optimized CRT (LOT-CRT) might confer additional benefit.

**Objectives:**

To compare echocardiographic and clinical characteristics between LB-CRT and LOT-CRT at a 6 month follow up.

**Materials and methods:**

This single center randomized controlled trial included patients with non-ischemic cardiomyopathy and left bundle branch block with left ventricular ejection fraction (LVEF) < 35% who underwent implantation of an atrial lead, a left bundle lead and a coronary sinus lead. Patients were randomized to LB-CRT or LOT-CRT 48 h after implant and followed-up for 6 months. LVEF, LV end systolic volume (LVESV), NYHA class, 6-min walk distance (6MWD) and response rates were compared between the two pacing modalities.

**Results:**

26 patients (12 in the LB-CRT group and 14 in the LOT-CRT group) were recruited in the study. The mean QRS duration of the population was 169.8 ± 20.6 ms and the mean LVEF was 21 ± 6.6%. Change in LVEF at 6 months (delta LVEF) in the LB-CRT group (15.7 ± 12.8%) was not significantly different from that in the LOT-CRT group (11.4 ± 14.2%; p = 0.43). Response rate in LB-CRT group (72.7%%), was comparable to that in the LOT-CRT group (71.4%; p = 0.94). LVESV, change in LVESV and 6MWD were also not significantly different between the two modalities.

**Conclusions:**

In patients with non-ischemic cardiomyopathy and LBBB, there was no additional benefit with LOT-CRT compared to LB-CRT.

## Graphical abstract

**Figure:**
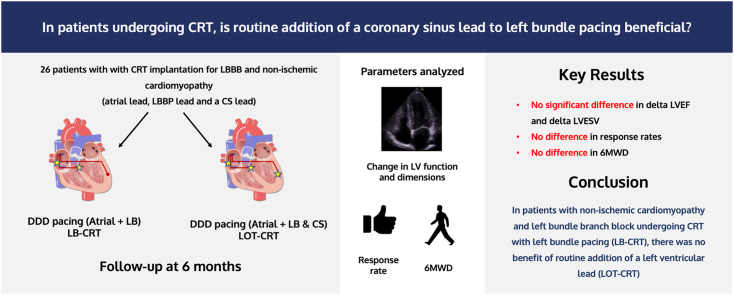

**Table:** Abbreviations:

ANOVA	Analysis of Variance
AV	Atrioventricular
CRT	Cardiac Resynchronization Therapy
CS	Coronary Sinus
ECG	Electrocardiogram
IEC	Institutional Ethics Committee
LBBB	Left Bundle Branch Block
LBBAP	Left Bundle Branch Area Pacing
LOT-CRT	Left Bundle Optimized Cardiac Resynchronization Therapy
LV	Left Ventricle
LVEF	Left Ventricular Ejection Fraction
NYHA	New York Heart Association
RV	Right Ventricle
V6RWPT	V6 R Wave Peak Time
VV	Ventriculo-ventricular
XML	Extensible Markup Language

## 1 Introduction

Cardiac resynchronization therapy (CRT) is an established treatment for patients with left ventricular (LV) systolic dysfunction and ventricular dyssynchrony. Conventional biventricular CRT utilizes two leads for ventricular pacing - one in the right ventricle (RV) and one in the coronary sinus (CS). Limitations of biventricular CRT include a lack of response in around one third of the patients [1], dyssynchrony related to myocardial pacing with the RV lead, non-physiological epicardial LV pacing, and suboptimal LV lead position in patients with unfavorable cardiac venous anatomy and scars [2]. Recent data has shown that left bundle branch area pacing (LBBAP) is an alternative to provide cardiac resynchronization and might confer additional benefits (LB-CRT) [3,4]. However, LB-CRT alone might not restore physiological activation of the left ventricle in patients with distal and/or diffuse conduction system disease. In these patients, addition of a lead in the coronary sinus, termed Left Bundle Optimized CRT (LOT-CRT) may result in better resynchronization. LOT-CRT has been shown to result in lesser QRS width and superior hemodynamic profile when compared to LB-CRT, especially when the results of LB-CRT are suboptimal [5,6]. Observational studies have also shown better outcomes with LOT-CRT as compared to LB-CRT and biventricular CRT in terms of higher LV ejection fraction (LVEF) and lesser number of heart failure rehospitalizations [7,8]. However, there are no randomized studies comparing these two modalities of resynchronization. The aim of this study was to compare LB-CRT and LOT-CRT in patients with non-ischemic cardiomyopathy who are candidates for CRT implantation in a randomized controlled manner.

## 2 Methods

This was a single center, prospective, randomized controlled trial conducted between March 2022 and November 2024. The study was approved by the Institute ethics committee (Project number JIP/IEC/2021/060) and was prospectively registered with the clinical trials registry of India (Registration number CTRI/2022/09/045773). All patients provided written informed consent.

We included patients above 18 years of age with non-ischemic dilated cardiomyopathy (LVEF <35% and normal epicardial coronaries), left bundle branch block, QRS duration of more than 130 ms who underwent successful CRT implantation with three leads – an atrial lead, a left bundle lead and a coronary sinus lead. Patients not in sinus rhythm were excluded.

All the patients underwent block randomization 48 h after implant (block size of 4 patients) using computer generated random numbers after consenting to participate in the study. The two groups were as follows:

1. LB-CRT - DDD pacing with ventricular pacing from the left bundle lead alone
2. LOT-CRT - DDD pacing with ventricular pacing from left bundle and CS leads

The primary objective of the study was to compare the change in LVEF (delta LVEF) from time of implant to 6 months after implant between LB-CRT and LOT-CRT. The secondary objectives included comparison of QRS duration, responder rates, echocardiographic parameters including LVEF and LVESV and 6-min walk distance (6MWD) between LB-CRT and LOT-CRT.

CRT implantation was done using standard techniques. Usually a quadripolar or bipolar lead was placed in the coronary sinus (CS) first. A fixed helix, lumenless lead (3830 SelectSecure, Medtronic, Minneapolis, MN) was used for LBBAP. The lead was deployed through a non-deflectable sheath (C315, Medtronic, Minneapolis, MN). The LBBAP lead was connected to the RV port in the CRT device. Left bundle capture at implant was decided as per treating physician's judgement and was redefined objectively later by a blinded observer as follows:

a) A paced RBBB morphology (typical/atypical) in V1
b) Transition from non-selective left bundle capture to selective left bundle capture or left ventricular septal capture
c) A short V6 R wave peak time (V6RWPT) of <101 ms which remained constant with low and high output pacing
d) V6-V1 interpeak interval of >44 ms

Patients who met ≥2 criteria mentioned above were classified as having left bundle branch capture. Patients who had an RBBB morphology (typical/atypical) but did not meet the other criteria for left bundle capture were classified as having left ventricular septal pacing (LVSP). Patients with a deep septal lead position but who did not meet the above mentioned criteria were classified as having deep septal pacing (DSP).

Bipolar pacing was used from both the CS and the LBBAP lead. For quadripolar CS leads, the bipole for pacing was chosen based on threshold and phrenic nerve stimulation. Pacing output for both the LB lead and the CS lead was set to twice the pacing threshold. The sensed and paced atrioventricular (AV) delays were programmed to 90 ms and 120 ms respectively. Ventricular pacing was performed from LBBAP lead first with a delay of 20 ms to CS lead pacing in the LOT-CRT group. This was done to achieve near simultaneous activation of the left ventricular septal and lateral walls.

All the patients were followed up at 6 months. Response at 6 months was defined as clinical response (improvement in NYHA class by at least one class without any heart failure hospitalisation) and echocardiographic response (increase in LVEF by at least 5 % or decrease in LVESV by at least 15 %). Super-response was defined as an absolute improvement in LVEF by ≥ 20% or improvement of LVEF to >50%

### 2.1 Echocardiography protocol

Image acquisition and processing was done on Philips EPIQ 7 (Philips Healthcare, Andover, MA, USA) ultrasound Machine with S5-1 probe. LVEF and LVESV at baseline and at 6 months was calculated using the modified biplane Simpson's method. Echocardiography was performed by an operator who was blinded to the patient's pacing mode.

### 2.2 Electrocardiography protocol

Digital 12-lead ECGs were recorded in AAI mode and DDD mode at randomization and at 6 months. ECGs were recorded in an XML format on Biocare iE-12A machine (Shenzhen Biocare Bio-Medical Equipment Co. Ltd, China) with a high pass filter set at 0.5 Hz, low pass filter set at 100 Hz and a sampling rate of 1000 Hz. All the measurements were done using digital callipers in an online XML ECG viewer [9]. An averaged single beat displayed in the 12x1 display format was used to make the measurements. QRS duration was measured using the global QRS method, i.e. from the earliest onset of the QRS in any of the 12 simultaneously recorded standard ECG leads to the latest QRS end in any of the 12 simultaneously recorded leads. QRS onset was defined as the beginning of the first rapid deflection.

## 3 Statistical analysis

We assumed a 20% increase in LVEF with LOT-CRT, 5% more than that reported with LBBAP in a previous study. Using the formula for sample size estimation for unpaired means and assuming a power of 80% and a 25% loss due to withdrawal and loss to follow-up, the sample size estimated was 40 patients. Continuous data was expressed as mean and standard deviation. Normality of data was confirmed using the Shapiro-Wilk test. Paired comparisons were made using a paired *t*-test. Comparison of more than 2 dependent variables was done by Repeated Measures ANOVA test. All the statistical analysis was carried out at 5% level of significance and a p value ≤ 0.05 was considered significant.

## 4 Results

**Fig. 1:**
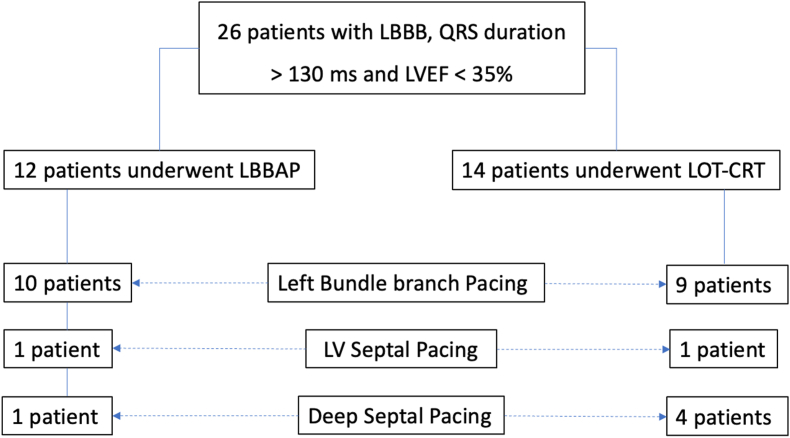
– Classification of the study population according to the type of left bundle capture.

A total of 26 patients (12 in the LB-CRT group and 14 in the LOT-CRT group) were recruited in the study over a period of 18 months. The study was stopped before the planned sample size was reached due to slow recruitment. Demographic and procedural characteristics of the patients are shown in Table 1. Mean age was 53.7 ± 8.4 years and 38.4% patients were female. NYHA III/IV symptoms were present in 92.3% of patients. ECG met the Strauss criteria for left bundle branch block in all the patients and the mean QRS duration was 169.8 ± 20.6 ms. Mean ejection fraction was 21 ± 6.6% prior to device implantation. Using the aforementioned criteria, left bundle branch capture was seen in 19 (73%) patients, LVSP in 2 (7.6%) patients and DSP in 5 (19.2%) patients(Fig. 1).

**Table 1:** Baseline characteristics.

		Entire population n = 26	Group I (LB-CRT) n = 12	Group II (LOT-CRT) n = 14	Group I vs Group II (p value)
Age (years)		53.5 ± 9.9	55.7 ± 8.3	52.1 ± 8.5	0.27
Females		45.8% (7)	50% (6)	28.5% (4)	0.26
NYHA Class	I	0	0%	0%	
	II	7.6% (2)	16.6% (2)	0%	0.11
	III	57.6% (15)	33.3% (4)	78.6% (11)	0.01
	IV	34.6% (9)	50% (6)	21.4% (3)	0.12
LBBB as per Strauss criteria		100% (26)	100% (12)	100% (14)	
QRS duration at baseline (ms)		169.8 ± 20.8	165.1 ± 14	173.5 ± 24.8	0.35
Paced QRS duration (ms)		131.1 ± 16.3	130.8 ± 15.9	131.4 ± 17.5	0.94
Paced QRS duration (from pacing spike; in ms)			151.6 ± 15.2	145.5 ± 10.4	0.38
LVEF (%)		23.7 ± 9	22.4 ± 7.6	19.7 ± 5.6	0.31
LVESV (ml)		152.8 ± 99.7	137.8 ± 62.6	176 ± 79.9	0.19

NYHA – New York Heart Association.

LBBB – Left Bundle Branch Block.

LVEF – Left Ventricular Ejection Fraction.

LVESV – Left Ventricular End-Systolic Volume.

**Fig. 2:**
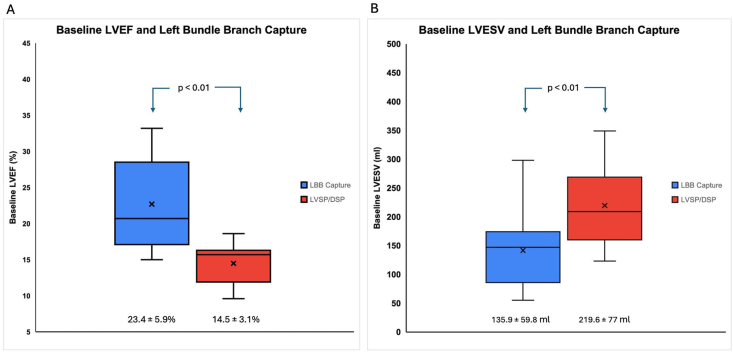
– Comparison of change in left ventricular ejection fraction (LVEF) at follow-up (delta LVEF) between LB-CRT and LOT-CRT.

Out of 26 patients, 25 patients completed follow-up. One patient expired three months after CRT implantation. No lead macro-dislodgement or phrenic nerve stimulation were seen during follow up in any patient. Change in LVEF from implant to 6 months (delta LVEF) was the primary outcome of the study. Delta LVEF in the LB-CRT group was 15.7 ± 12.8%, whereas it was 11.4 ± 14.2% in the LOT-CRT group (p = 0.43, Fig. 2). Change in LVESV (Table 2) was also not significantly different between the two groups (p = 0.70). Significant improvement in LVEF and LVESV was seen at 6 months in both the groups, but there were no significant differences between the two modalities (Table 2).

**Table 2:** Comparison of Left Ventricular Ejection Fraction (LVEF) and end-systolic volume (LVESV) in LB-CRT and LOT-CRT groups.

	LB-CRT (n = 12)	LOT-CRT (n = 14)	LB-CRT vs LOT-CRT (p value)
LVEF at baseline	22.4 ± 7.6%	19.7 ± 5.6%	0.32
LVEF at follow-up	37.2 ± 15.9%	31.1 ± 14.5%	0.33
Delta LVEF	15.7 ± 12.8%	11.4 ± 14.2%	0.43
LVESV at baseline	137.8 ± 62.7 ml	176 ± 79.9 ml	0.19
LVESV at follow-up	95.3 ± 55.1 ml	117. 3 ± 73.3 ml	0.42
Delta LVESV	49.3 ± 60.0 ml	58.6 ± 62.6 ml	0.71

All the patients who completed the 6-month follow up showed clinical response (defined as improvement in NYHA scale by at least one class). Echocardiographic response to CRT, which was defined as a 5% increase in LVEF or 15% reduction in LVESV, was seen in 75% of the patients (19/25). The response rate in LB-CRT group (72.7%%), was not significantly different than that in the LOT-CRT group (71.4%; p = 0.94). Echocardiographic super-response was seen in 36.3% (4/11) patients in the LB-CRT group and 21.4% (3/14) patients in the LOT-CRT group. The mean 6MWD at six months follow up with LB-CRT (348.7 ± 39.2 m) was not significantly different from that with LOT-CRT (337.2 ± 64.9 m, p = 0.61).

Among patients with confirmed left bundle branch capture, there were no significant differences in delta LVEF, delta LVESV, responder and super-responder rates and 6MWD between the LB-CRT and LOT-CRT groups (Table 3).

**Table 3:** Comparison of Left Ventricular Ejection Fraction (LVEF) and end-systolic volume (LVESV) of LB-CRT and LOT-CRT groups in patients with left bundle branch capture.

	LB-CRT (n = 10)	LOT-CRT (n = 9)	LB-CRT vs LOT-CRT (p value)
Delta LVEF	15.7 ± 12.8%	11.4 ± 14.2%	0.43
Delta LVESV	49.3 ± 60.0 ml	58.6 ± 62.6 ml	0.71
Response rate	6/9 (66.6%)	6/9 (66.6%)	1.00
Super-response rate	3/9 (33.3%)	2/9 (22.2%)	0.59
6MWD	350.5 ± 39.6	351.3 ± 65.5	0.97

**Fig. 3:**
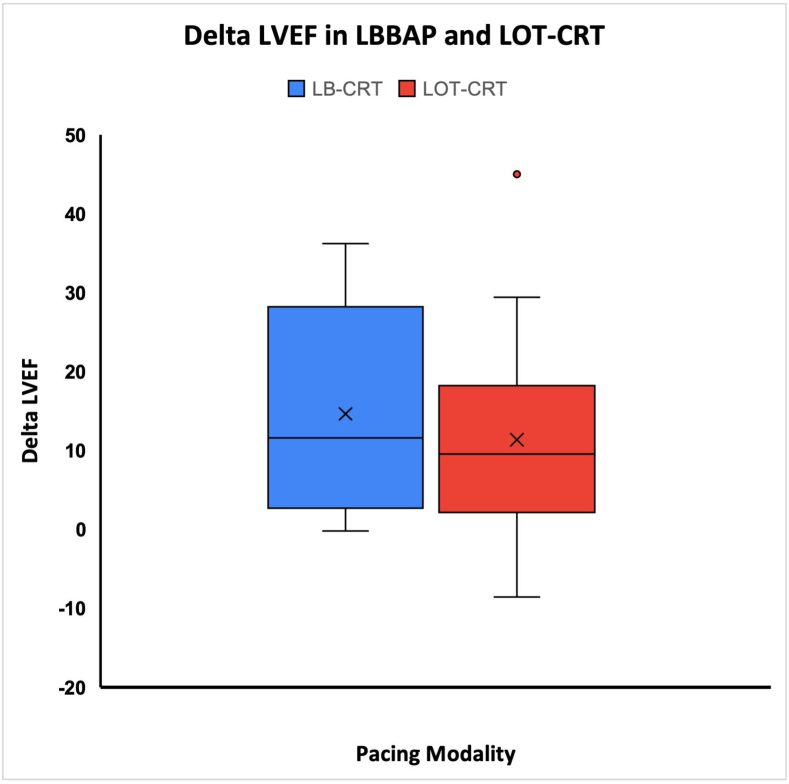
– Left ventricular ejection fraction (LVEF) and left ventricular end-systolic volume (LVESV) at baseline in patients with and without left bundle branch capture.

**Fig. 4:**
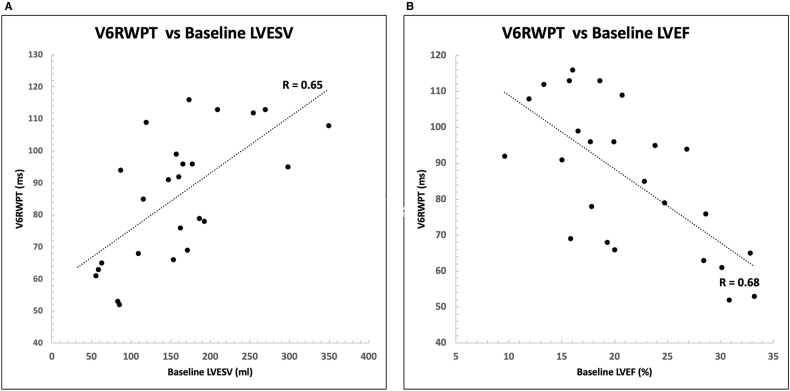
– Correlation of V6 R-wave peak time (V6RWPT) at implant with baseline left ventricular left ventricular end-systolic volume (LVESV, Panel A) and left ventricular ejection fraction (LVEF, Panel B).

Patients in whom left bundle branch capture could not be demonstrated had significantly lower LVEF and higher LVESV at baseline (Fig. 3). There was a significant correlation between V6 R wave peak time (V6RWPT) values during left bundle lead implantation and baseline LVESV (r = 0. 65) and LVEF (r = 0.68) (Fig. 4). The extent of improvement in LV function (delta LVEF and delta LVESV) however did not correlate with the V6RWPT values (r = 0.15 and 0.38 respectively).

**Fig. 5:**
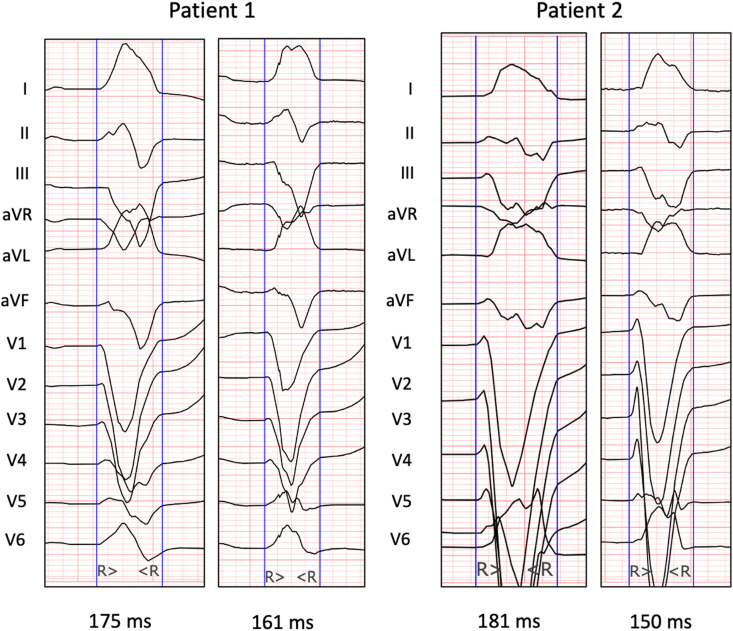
– Electrocardiograms of two patients with a reduction in native QRS duration at follow-up.

Native QRS duration at follow-up was available in 19 patients. A reduction in the native QRS duration of at least 5 ms at follow up, i.e. reverse electrical remodelling was seen in 9 patients (47%). Echocardiographic response was seen in 88.8% (8/9) of these patients. ECGs of two such patients have been shown in Fig. 5. An increase in the native QRS duration of at least 5 ms was seen in 4 patients (4.1%). None of these patients showed echocardiographic response. There change in QRS duration (delta QRSd) correlated weakly with delta LVEF (r = 0.34) and delta LVESV (r = 0.39).

## 5 Discussion

In this randomized controlled trial, we compared LB-CRT and LOT-CRT in patients with non-ischemic cardiomyopathy and LBBB. We found that the improvement in left ventricular function was not significantly different between the two groups at a 6-month follow-up. Additionally, there was no significant difference in the left ventricular dimensions, responder rate and 6MWD between LB-CRT and LOT-CRT.

There are theoretical reasons why LOT-CRT may provide better resynchronization. Conduction system disease distal to the pacing site and/or diffuse conduction system disease may be present in some patients and can result in sub-optimal resynchronization with left bundle pacing alone. It has been shown that V6RWPT, which is the ECG marker of the LV lateral wall activation time, is longer in patients with LBBB than in patients without LBBB, even with successful left bundle capture [10], suggesting that distal conduction disease is common. In these patients, the addition of a left ventricular lead (LOT-CRT) might result in better electrical resynchronization. A computational modelling study found that patients with diffuse left ventricular conduction system disease showed significantly shorter activation times with LOT-CRT when compared to LB-CRT [11]. LOT-CRT has also been shown to have better electrical resynchronization and better improvement in acute hemodynamic profile, especially when results of left bundle pacing are sub-optimal [5,6].

The lack of additional echocardiographic and clinical benefit with LOT-CRT compared to LB-CRT in our study can be attributed to several reasons. First, patients in the LOT-CRT group had a lower mean LVEF and higher QRS duration at baseline compared to the patients in the LB-CRT group, although this difference was not of statistical significance. This might have influenced the results of the study. Second, the benefit accrued by better electrical resynchronization with LOT-CRT may be too small to translate into clinically meaningful benefit in terms of improvement in LV function. Third, arbitrarily fixed AV and VV delays (90 and 20 ms respectively) and the lack of AV and VV delay optimisation to maintain uniformity in the study protocol may have influenced the results of the study. There is only one other study comparing LB-CRT and LOT-CRT. In this study, Jastrzębski et al. have compared electrical resynchronization with LOT-CRT and LB-CRT in a prospective non-randomized fashion [8]. However, the major reason behind LOT-CRT implantation in this study was a suboptimal result of LB-CRT or conventional CRT. The design was non-consecutive and criteria for selecting patients for LOT-CRT were not uniform. Only QRS duration was compared between the groups and the apparent benefit of an additional LV lead on the QRS duration was probably due to the criteria used for selecting patients for LOT-CRT. As a result, it is difficult to draw any reliable conclusions from this study.

Like previous studies, echocardiographic response in our study was defined as an increase in LVEF by 5% or decrease in LVESV by 15%. LB-CRT has been shown to have an echocardiographic response rate of around 75% and a super-response rates of around 30% in previous studies6. In the study by Jastrzebski et al. [8], echocardiographic response and super-response rates in patients undergoing LOT-CRT were 62.8% and 24.4% respectively. Echocardiographic response rates (72.7% with LB-CRT and 71.4% with LOT-CRT) and super-response rates (36.3% with LB-CRT and 21.4% with LOT-CRT) in our study are in general comparable with previously published studies.

Determination and classification of left bundle capture was done using the criteria described in EHRA clinical consensus statement7. Accordingly, LBBAP was successfully achieved in 80.7% (19/26) patients. This is similar to the LBBAP success rates in two recent studies (84.6% in the study by Chen et al. [12] and 83.5% in the study by Zhu et al. [13]). Moreover, patients in whom left bundle capture could not be demonstrated (LV septal/deep septal pacing) had significantly lower mean LVEF and higher mean LVESV at baseline. This validates the findings of a recent retrospective study by Graterol et al. [14], where it was found that a higher LV end diastolic diameter at baseline was more likely to result in an unsuccessful left bundle branch capture.

There were two interesting observations in the study population. The V6RWPT at implant were found to correlate well with the baseline LV dimensions and function. There was however no significant correlation between V6RWPT and the extent of improvement in LV function or reduction in LV volume. This phenomenon has also been recently described in a study by Chen et al. [15] in 83 patients undergoing LB-CRT. Our study lends support to these findings and underscores the need for using V6RWPT values indexed to baseline LV dimensions instead of using arbitrary cutoffs.

The other interesting finding was the presence of reverse electrical remodelling seen in patients undergoing LB-CRT/LOT-CRT. Close to one-half patients showed a reduction in the native QRS duration of at least 5 ms at follow-up. 90% of these patients showed echocardiographic response. Among patients with an increase in native QRS duration at follow-up, none showed echocardiographic response. Reverse electrical remodelling has been described in the context of conventional biventricular CRT [16,17]. With regards to conduction system pacing, literature about reverse electrical remodelling is limited to a single case report [18]. To our knowledge, this is the first study to describe this phenomenon and it deserves further attention.

### 5.1 Strengths

The main strength of the study is its ‘all comer’ nature - all patients underwent implantation of a left bundle lead and a CS lead irrespective of the left bundle paced QRS duration, thus eliminating selection bias. The patient population was homogeneous with all patients having non-ischemic cardiomyopathy and LBBB. Clinically important endpoints like improvement in left ventricular function and 6-min walk distance were studied.

### 5.2 Limitations

This was a single centre study with a small sample size. The planned sample size was not reached due to slow recruitment and this might have introduced a type II error. Left bundle capture was analyzed only at implant and not at follow-up. Consequently, information about lead micro-dislodgement is not available. The follow up period in the study was only 6 months. While most improvement after CRT is seen in this time period, further improvement in LV function is likely to occur after this. Aforementioned reasons could have resulted in small benefits of LOT-CRT not being identified in this study. The AV and VV delays were arbitrarily decided and same for all patients to maintain uniformity in the study protocol.

## 6 Conclusions

In this small randomized controlled trial of patients with non-ischemic cardiomyopathy and left bundle branch block undergoing CRT with left bundle pacing alone (LB-CRT) or in combination with left ventricular pacing (LOT-CRT), there were no differences in clinical and echocardiographic outcomes between LB-CRT and LOT-CRT.

## Informed consent

All patients provided written informed consent.

## Approval of the research protocol

The study was approved by the Institute ethics committee (Project number JIP/IEC/2021/060, Date of approval 02/082021)

## Registry and the registration no. of the study/trial

This trial was prospectively registered with the clinical trials registry of India (Registration number: CTRI/2022/09/045773, Date of Registration: 22/09/2022).

## Data availability statement

The data that support the findings of this study are available from the corresponding author upon reasonable request.

## Animal studies

N/A.

## Declaration of competing interest

The authors declare that they have no known competing financial interests or personal relationships that could have appeared to influence the work reported in this paper.
